# Hirschsprung disease and hepatoblastoma: case report of a rare association: Doença de Hirschsprung e hepatoblastoma: relato de caso de uma associação rara

**DOI:** 10.1590/1516-3180.2014.9200311

**Published:** 2015-10-09

**Authors:** Raquel Borges Pinto, Ana Regina Lima Ramos, Ariane Nadia Backes, Beatriz John dos Santos, Valentina Oliveira Provenzi, Mário Rafael Carbonera, Maria Lúcia Roenick, Pedro Paulo Albino dos Santos, Fabrizia Falhauber, Meriene Viquetti de Souza, João Vicente Bassols, Osvaldo Artigalás

**Affiliations:** I MD. Physician, Department of Pediatric Gastroenterology, Hospital da Criança Conceição, Grupo Hospitalar Conceição (GHC), Porto Alegre, Rio Grande do Sul, Brazil.; II MD. Physician, Department of Pediatric Surgery, Hospital da Criança Conceição, Grupo Hospitalar Conceição (GHC), Porto Alegre, Rio Grande do Sul, Brazil.; III MD. Physician, Department of Pathological Anatomy, Hospital Nossa Senhora da Conceição, Grupo Hospitalar Conceição (GHC), Porto Alegre, Rio Grande do Sul, Brazil.; IV MD. Resident, Department of Pediatric Surgery, Hospital da Criança Conceição, Grupo Hospitalar Conceição (GHC), Porto Alegre, Rio Grande do Sul, Brazil.; V MD. Physician, Department of Pediatric Oncology and Hematology, Hospital da Criança Conceição, Grupo Hospitalar Conceição (GHC), Porto Alegre, Rio Grande do Sul, Brazil.; VI MD. Resident, Department of Pediatrics, Hospital da Criança Conceição, Grupo Hospitalar Conceição (GHC), Porto Alegre, Rio Grande do Sul, Brazil.; VII MD. Physician, Department of Medical Genetics, Hospital da Criança Conceição, Grupo Hospitalar Conceição (GHC), Porto Alegre, Rio Grande do Sul, Brazil.

**Keywords:** Hirschsprung disease, Hepatoblastoma, Intestinal atresia, Hearing loss, sensorineural, Cataract

## Abstract

**CONTEXT:**

Hirschsprung disease is a developmental disorder of the enteric nervous system that is characterized by absence of ganglion cells in the distal intestine, and it occurs in approximately 1 in every 500,000 live births. Hepatoblastoma is a malignant liver neoplasm that usually occurs in children aged 6 months to 3 years, with a prevalence of 0.54 cases per 100,000.

**CASE REPORT:**

A boy diagnosed with intestinal atresia in the first week of life progressed to a diagnosis of comorbid Hirschsprung disease. Congenital cataracts and sensorineural deafness were diagnosed. A liver mass developed and was subsequently confirmed to be a hepatoblastoma, which was treated by means of surgical resection of 70% of the liver volume and neoadjuvant chemotherapy (ifosfamide, cisplatin and doxorubicin).

**CONCLUSION:**

It is known that Hirschsprung disease may be associated with syndromes predisposing towards cancer, and that hepatoblastoma may also be associated with certain congenital syndromes. However, co-occurrence of hepatoblastoma and Hirschsprung disease has not been previously described. We have reported a case of a male patient born with ileal atresia, Hirschsprung disease and bilateral congenital cataract who was later diagnosed with hepatoblastoma.

## RESUMO

**CONTEXTO:**

A doença de Hirschsprung é uma desordem do desenvolvimento do sistema nervoso entérico, que é caracterizada pela ausência de células ganglionares no intestino distal, ocorrendo em cerca de 1 a cada 500.000 nascimentos. O hepatoblastoma é uma neoplasia maligna do fígado que geralmente ocorre em crianças de 6 meses a 3 anos, com prevalência de 0,54 casos por 100.000.

**RELATO DE CASO:**

Um menino com diagnóstico de atresia intestinal na primeira semana de vida evoluiu com diagnóstico concomitante de doença de Hirschsprung. Catarata congênita e surdez neurossensorial foram diagnosticadas. Surgiu lesão hepática com posterior confirmação de hepatoblastoma, tratado com ressecção cirúrgica de 70% do volume hepático e quimioterapia neoadjuvante (ifosfamida, cisplatina e doxorubicina).

**CONCLUSÃO:**

Sabe-se que a doença de Hirschsprung pode estar associada a síndromes de predisposição ao câncer, da mesma forma que o hepatoblastoma já foi correlacionado a certas síndromes congênitas malformativas. No entanto, até o momento, a associação de hepatoblastoma com a doença de Hirschsprung não foi descrita. Relatamos o caso de um menino que nasceu com atresia ileal, doença de Hirschsprung, catarata congênita bilateral e com posterior diagnóstico de hepatoblastoma.

## INTRODUCTION

Hirschsprung disease is an unusual, but well-recognized cause of chronic constipation in children. It occurs in approximately 1 in every 500,000 live births, and most commonly presents as a neonatal bowel obstruction. However, in older children, it may present as chronic constipation or enterocolitis.^1^ Hirschsprung disease occurs as an isolated trait in 70% of the patients, is associated with a chromosomal abnormality in 12% and occurs with additional congenital anomalies in 18%.^2^

Primary hepatic malignancies account for approximately 1% of cancers in children, and can be divided into two major histological subgroups: hepatoblastoma and hepatocellular carcinoma.^3^ The overall prevalence of hepatoblastoma is 0.54 per 100,000 individuals, and it occurs primarily in children younger than 5 years of age.^4^ In Brazil, the median age-adjusted incidence rate (AAIR) of hepatoblastoma ranged from 0.0 to 2.8 per million in a study that included data from 13 cities; notably, the highest incidence was found in our city (Porto Alegre), with a median AAIR of 2.78.^5^ We report a case of hepatoblastoma in a child previously diagnosed with ileal atresia and Hirschsprung disease, which is an unusual association.

## CASE REPORT

**Figure 1.**
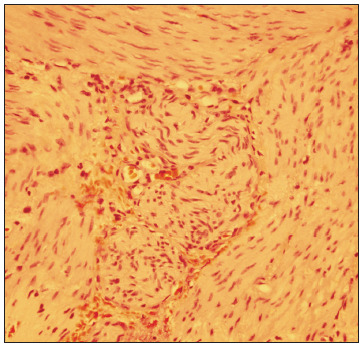
Hirschsprung disease: myenteric plexus lacking ganglion cells (hematoxylin-eosin staining, 400 x).

A male infant was born after 28 weeks of gestation with a birth weight of 910 grams and Apgar scores of 4 at the first minute and 7 at the fifth minute. At 2 days of age, still without bowel movements, he developed abdominal distension and vomiting. Abdominal radiography showed severe small-bowel distension and wall edema without pneumoperitoneum. Oral feeding was discontinued and antibiotics and total parenteral nutrition were started due to clinical suspicion of necrotizing enterocolitis. A barium enema revealed a state of microcolon due to disuse. On laparotomy, intestinal atresia in the terminal ileum and a disconnected cecum were identified. Ileostomy and cecostomy were performed, and a set of biopsies was obtained, going from the transverse colon to the rectum. Histopathological examination revealed absence of ganglion cells in the rectum and sigmoid colon, consistent with Hirschsprung disease (Figure 1). A Duhamel procedure was performed, with total colectomy due to absence of ganglion cells throughout the colon, which was identified during frozen section examination.

During a routine physical examination, the patient was diagnosed with congenital cataracts, which were surgically corrected. Genetic evaluation was normal and TORCH (*Toxoplasma gondii*, rubella, cytomegalovirus, herpes simples and other viruses) complex screening was negative. An echocardiogram showed a large patent ductus arteriosus with hemodynamic impairment. Indomethacin treatment was unsuccessful, and surgical closure was performed. Furthermore, the patient was evaluated by a speech therapist and deafness was detected.

**Figure 2.**
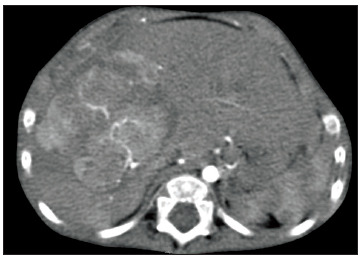
Computed tomography (CT) scan of the abdomen. A space-occupying lesion is visible in the liver, located between the middle and right hepatic veins (segment VIII).

**Figure 3.**
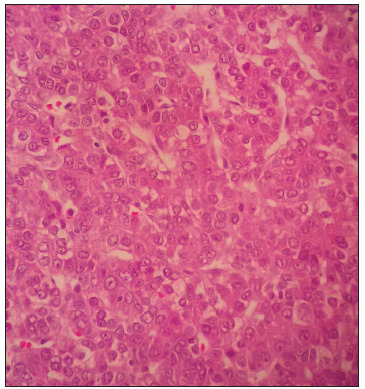
Hepatoblastoma, epithelial type (hematoxylin-eosin staining, 400 x).

**Figure 4.**
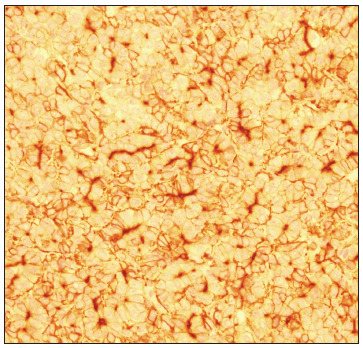
Hepatoblastoma immunohistochemistry: positive for carcinoembryonic antigen (CEA), with canalicular pattern, 400 x.

**Figure 5.**
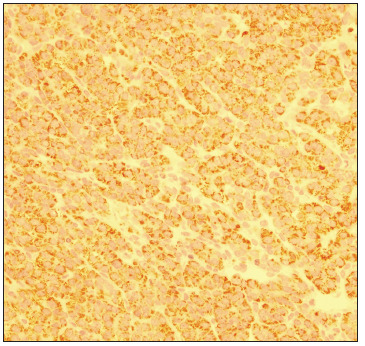
Hepatoblastoma immunohistochemistry: cytoplasmic positivity for Hep Par1, with granular pattern, 400 x.

At the age of 25 months, during computed tomography (CT) on the chest to evaluate a lung malformation, a tumor in the right hepatic lobe measuring 5.6 cm x 4.3 cm, with marked contrast uptake, was incidentally observed (Figure 2). A liver biopsy was performed, and subsequent immunohistochemical examination of the biopsy specimen revealed epithelial-type hepatoblastoma (Figures 3, 4 and 5). The patient was started on neoadjuvant chemotherapy with ifosfamide, cisplatin and doxorubicin (four cycles). At that time, the alpha-fetoprotein (AFP) level was 8229 ng/ml (reference range: < 10 ng/ml). An abdominal CT scan performed after chemotherapy showed a significant reduction in tumor volume (3.2 cm x 2.6 cm). Right hepatectomy was performed, leaving a residual liver of 30%, followed by two cycles of adjuvant chemotherapy. Currently, the patient is asymptomatic, with no evidence of tumor recurrence, he has normal bowel movements and normal AFP level (2.79 ng/ml).

## DISCUSSION

Hirschsprung disease is a developmental disorder of the enteric nervous system that is characterized by absence of ganglion cells in the myenteric (Auerbach’s) and submucosal (Meissner’s) plexuses of the distal intestine, which results in lack of peristalsis and functional intestinal obstruction.^6^ In 80-85% of the cases, the aganglionic region is limited to the rectum and sigmoid colon, as in our patient.^7^

The heterogeneous nature of Hirschsprung disease seems to be supported by evidence of mutations in a variety of genes. The most commonly identified gene is the *RET* proto-oncogene, which is commonly found in familial and long-segment disease. It remains unclear how these mutations result in aganglionosis, but there is some evidence that early neuronal cell death may be a prominent mechanism. Hirschsprung disease is associated with a variety of other congenital abnormalities: malrotation, genitourinary abnormalities, congenital heart disease, limb abnormalities, mental retardation and dysmorphic features.^6^ Many of these patients also have other abnormalities of neural crest-derived tissues, such as pigmentation disorders and sensorineural deafness, including Waardenburg syndrome.^6^ However, the association of Hirschsprung disease with profound congenital deafness in the absence of other syndromic features, as in our patient, has been reported before.^8^^,^^9^ In the present case, our patient presented with sensorineural deafness and bilateral congenital cataracts, but no pigmentation disorders.

Hirschsprung disease may also be associated with syndromes predisposing towards cancer, such as familial medullary thyroid carcinoma, multiple endocrine neoplasia type 2A and type 2B and neuroblastoma. A review of the literature was conducted through an online search for the MeSH, EMTREE and MeSH/DeCS terms “Hirschsprung disease” and “hepatoblastoma” in PubMed, Embase (via Elsevier) and LILACS (via Bireme), respectively (Table 1), but did not find any previous reports of comorbid Hirschsprung disease and hepatoblastoma. On the other hand, hepatoblastoma may also be associated with certain congenital syndromes (such as Beckwith-Wiedemann syndrome and trisomy 18 syndrome).^10^^,^^11^

**Table 1.**
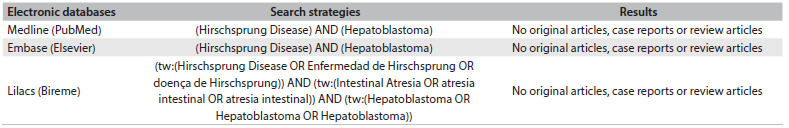
Database search results for Hirschsprung disease and hepatoblastoma on August 6, 2014

The incidence of hepatoblastoma in the United States (2.2 cases per 1 million children aged 0-14 years, over the period 2006-2010^12^) appears to have doubled over recent decades.^13^ The cause of this increase in incidence is unknown, but it may be related to increasing survival of very low birth weight premature infants.^14^ In Brazil, there have been very few cases, and they are recorded in only 8 of the 14 population-based cancer registries. The incidence appears to be highest in the central-western region of the country.^14^^,^^15^ The patient in this case report met the criteria for the highest risk of hepatoblastoma (male, white and extremely premature, with birth weight < 1 kg).^16^^,^^17^

One sensitive but nonspecific biomarker for the presence of hepatoblastoma is AFP. This is a useful clinical marker for monitoring treatment effectiveness and tumor recurrence, since 90% of the patients at diagnosis have highly elevated serum levels of AFP.^11^ Because the liver has excellent regeneration capacity, up to 80% of this organ can be resected.^3^ The goal of therapy for hepatoblastoma is complete surgical resection^3^ (which was the result achieved in the case reported here), because the majority of patients survive if a hepatoblastoma is removed completely. The overall 5-year survival rate for children with hepatoblastoma is 70%.^12^ Metastases are found in approximately 20% of patients at diagnosis (usually in the lungs, central nervous system (CNS) and eyes).^15^

## CONCLUSION

We have reported a case of an unusual association of hepatoblastoma in a child with previous diagnoses of Hirschsprung disease, ileal atresia, deafness and cataracts. Complete resection of the tumor was achieved, with favorable clinical evolution. We emphasize the importance of comprehensive assessment of patients with Hirschsprung disease, due to the possibility of several chromosomal abnormalities and associated congenital anomalies.
